# Investigating a New Generation of Ribozymes in Order to Target HCV

**DOI:** 10.1371/journal.pone.0009627

**Published:** 2010-03-10

**Authors:** Michel V. Lévesque, Dominique Lévesque, Francis P. Brière, Jean-Pierre Perreault

**Affiliations:** Département de Biochimie, Faculté de Médecine et des Sciences de la santé, Université de Sherbrooke, Sherbrooke, Québec, Canada; Pohang University of Science and Technology, Republic of Korea

## Abstract

For a long time nucleic acid-based approaches directed towards controlling the propagation of Hepatitis C Virus (HCV) have been considered to possess high potential. Towards this end, ribozymes (i.e. RNA enzymes) that specifically recognize and subsequently catalyze the cleavage of their RNA substrate present an attractive molecular tool. Here, the unique properties of a new generation of ribozymes are taken advantage of in order to develop an efficient and durable ribozyme-based technology with which to target HCV (+) RNA strands. These ribozymes resulted from the coupling of a specific *on*/*off* adaptor (SOFA) to the ribozyme domain derived from the Hepatitis Delta Virus (HDV). The former switches cleavage activity “*on*” solely in the presence of the desired RNA substrate, while the latter was the first catalytic RNA reported to function naturally in human cells, specifically in hepatocytes. In order to maximize the chances for success, a step-by-step approach was used for both the design and the selection of the ribozymes. This approach included the use of both bioinformatics and biochemical methods for the identification of the sites possessing the greatest potential for targeting, and the subsequent in vitro testing of the cleavage activities of the corresponding SOFA-HDV ribozymes. These efforts led to a significant improvement in the ribozymes' designs. The ability of the resulting SOFA-HDV ribozymes to inhibit HCV replication was further examined using a luciferase-based replicon. Although some of the ribozymes exhibited high levels of cleavage activity in vitro, none appears to be a potential long term inhibitor in cellulo. Analysis of recent discoveries in the cellular biology of HCV might explain this failure, as well as provide some ideas on the potential limits of using nucleic acid-based drugs to control the propagation of HCV. Finally, the above conclusions received support from experiments performed using a collection of SOFA-HDV ribozymes directed against HCV (−) strands.

## Introduction

Hepatitis C Virus (HCV) is the major worldwide cause of both blood transfusion-associated and sporadic non-A, non-B hepatitis. Estimates place the number of HCV-infected individuals worldwide at 170 million, representing nearly 3% of the world's population [1], [2]. In 20–30% of these patients the HCV infection is acute and the virus is naturally cleared. The majority (70–80%) remain chronically infected, leading to various clinical outcomes including an asymptomatic carrier state with normal or almost normal liver functions, acute hepatitis and, about 50% of the time, chronic hepatitis. Today, chronic HCV infections can only be treated with pegylated interferon-α (IFN-α) [1], [2]. Unfortunately, about 70% of compliant patients experience a relapse, and only 25% maintain low serum alanine aminotransferase levels. Although a combination therapy including the purine nucleoside analogue ribavirin improves the rate of success, only 40% of patients infected with HCV achieve a sustained response. Moreover, in clinical practice many patients do not qualify for IFN-α therapy for several reasons. Hence, only a minority of patients with chronic hepatitis C can be successfully treated and, consequently, projections indicate that the mortality rate from hepatocellular carcinoma associated with chronic hepatitis C will further increase in the next 15–20 years [1], [3]. Clearly, it is an urgent mission to search for innovative strategies with which to treat/cure HCV infected patients.

**Figure 1:**
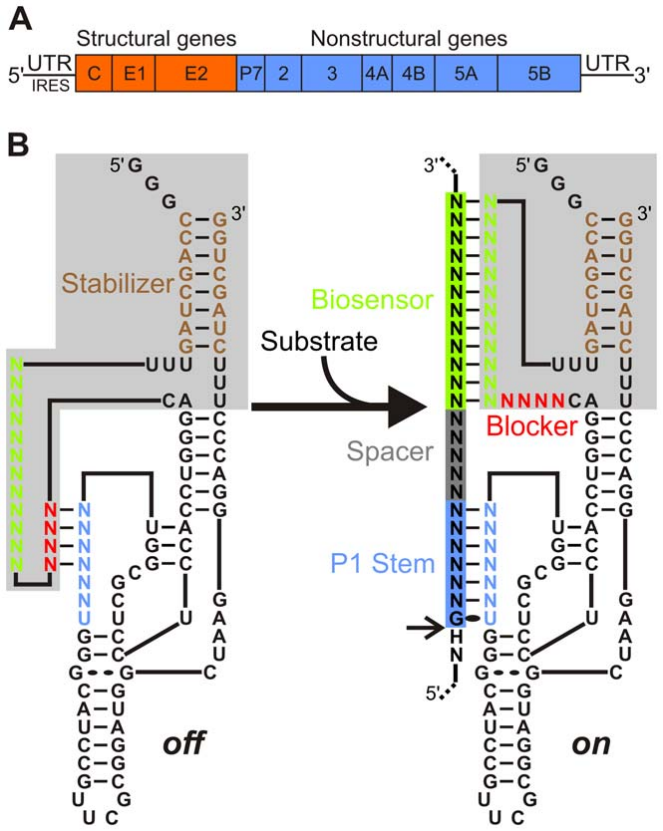
Schematic representations of both the HCV genome and the SOFA-HDV ribozyme. (A) HCV genomic RNA organization including the structured 5′ and 3′ UTRs. (B) Secondary structure of both the *off* (left) and *on* (right) conformations of the SOFA-HDV ribozyme. The SOFA module is highlighted in grey. The P1 domain (ribozyme binding domain), the biosensor, the blocker and the stabilizer domain are indicated blue, green, red and brown, respectively. The spacer region separating the substrate' sequences binding by the P1 and biosensor domains of the ribozyme is indicated in grey. The arrow indicates the cleavage site. All structures have been previously described [29], [30].

HCV, a member of the *Flaviviridae* family, is an enveloped virus with a plus-stranded RNA genome ∼9,6 kb in length (Figure 1A) [4]. Six major genotypes and more than 50 minor subtypes have be distinguished. The high replicative activity of the virus, together with the lack of a proofreading function of the viral RNA polymerase, provides the basis for the significant genetic variability of HCV. This genome carries a single long open reading frame (ORF) which encodes a polyprotein of ∼3,010 amino acids that is subsequently cleaved both co- and post-translationally into the mature viral proteins. The ORF is flanked by structured 5′ and 3′ untranslated regions (UTRs) that are important for both the translation of the polyprotein and the replication of the genome. For example, the 5′ UTR is highly conserved among different HCV isolates and contains an internal ribosomal entry site (IRES) essential for the cap-independent translation of the viral RNA [5].

Several antiviral strategies are being explored for the treatment of HCV infections [6], [7]. For example, antiviral inhibitors that block essential viral enzymes, such as the NS3–4 protease or the NS5B polymerase, have led to the discovery of several compounds that are currently at different stages of development [8]–[10]. Moreover, gene-inactivation approaches, including the use of antisense oligonucleotides and, more recently, RNA interference (RNAi), also show some promise in terms of being able to inhibit both HCV replication and gene expression in experimental systems [11]–[15]. However, the RNAi approach has been shown to trigger an immunological response, which poses an important limitation in terms of further therapeutical applications [16]–[18]. Ribozymes (Rz), RNA molecules that catalyze the cleavage of RNA substrates, are an interesting alternative to the RNAi approach to gene inactivation. Both ribozymes and deoxyribozymes have been demonstrated to block both viral replication and gene expression in experimental systems both in vitro and in cellulo [19]–[22].

Among the different ribozymes, Hepatitis Delta Virus ribozyme (HDV Rz) was for a long time the sole example derived from an RNA species naturally found in human cells (i.e. the infected hepatocytes) [23], [24]. As a result, HDV Rz offers several unique properties as a potential tool, including the natural ability to function in the presence of human proteins and at the physiological magnesium concentration (1 mM Mg^2+^). Furthermore, HDV Rz has a long half-life, regardless of both the cell line tested and the means of transfection used [23]. Thus, HDV Rz appears to be well adapted to the human cell environment and is therefore an interesting potential candidate for the development of a gene-inactivation system. This potential has subsequently been demonstrated in vitro as well as in cellulo by the use of HDV ribozyme to cleave various natural mRNA [24]–[28]. The HDV Rz folds into a model pseudoknot secondary structure in which substrate recognition is based solely on the formation of the P1 stem, a stem that involves only 7 base pairs (bp) (Figure 1B). Recently, molecular engineering has led to the development of a novel target-dependent riboswitch that increases HDV Rz fidelity [29]. This latter ribozyme possesses a specific *on/off* adapter (SOFA) that switches the cleavage activity from *off* (i.e. like a “safety lock”) to *on* solely in the presence of the desired RNA substrate. The SOFA module is composed of three domains: a blocker, a biosensor and a stabilizer [30]. The blocker sequence inhibits the cleavage activity of the ribozyme by binding, in *cis*, the P1 domain of the ribozyme portion. This provides an inactive SOFA-HDV ribozyme, i.e. *off* conformation. The biosensor must bind its complementary sequence on the substrate in order to unlock the SOFA module, thereby permitting the folding of the catalytic core into the *on* conformation. Both the blocker and the biosensor have been shown to increase the substrate specificity of the ribozyme's cleavage by several orders of magnitude compared with the wildtype HDV ribozyme [29]. This is due mainly to the addition of the biosensor domain that increases the binding strength of the HDV ribozyme to its target but it is also due to the fact that the blocker domain interacts with the P1 domain and decreases its binding capacity. It is important to note that the sequences of the substrate binding both the P1 and the biosensor domains are not contiguous, but rather separated by a small region named spacer that usually varies from 4 to 7 nt for optimal design [30]. Finally, the presence of the stabilizer (i.e. the stem that brings together both the 5′ and 3′ extremities), which has no effect on the cleavage activity, stabilizes the SOFA-HDV ribozymes in vivo against ribonucleases [29]. This new development provides a highly specific and improved HDV-based molecular tool that displays significant potential for application in the fields of both functional genomics and gene therapy.

In the present work, the unique properties of the HDV Rz that are improved by the presence of the SOFA adapter are used in order to attempt to develop a ribozyme-based technology with which to target HCV. Sites with greatest potential for targeting in the HCV strands of both polarities were identified, followed by a screen to find the most active SOFA-HDV ribozymes.

## Results

### Identification of Potential Target Sites in the HCV (+) Strand RNA

Since HCV is known to exhibit several genotypes and many subgenotypes, the identification of the most highly conserved sequences within its genome is critical to the development of nucleic acid based drugs that possess the ability to specifically bind to most, if not all, of the sequence variants. Consequently, only the first 341 nucleotides (nt) of the HCV (+) strand that correspond to the 5′ UTR, a region that is known to be highly conserved, were considered for targeting. Moreover, only the HCV (+) strand was analyzed because it has been reported to be significantly more abundant then its (−) counterpart [31], [32].

**Figure 2:**
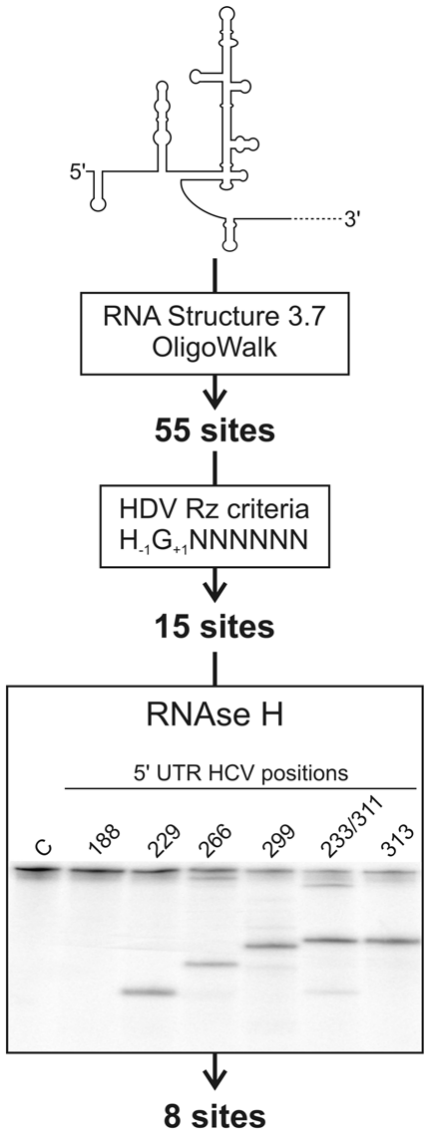
Schematic representation of the 3-step procedure used for the identification of the sites possessing the greatest targeting potential in the HCV 5′-UTR. Step 1 involved a bioinformatic analysis that included both the prediction of the secondary structure and the identification of the 7 nt streches most likely to be bound by the ribozyme's P1 region using both the RNA structure 3.7 and Oligowalk softwares. Step 2 involved the selection of the sequences that fulfill the HDV ribozyme requirements. Step 3 involved the RNase H hydrolysis assay. The autoradiogram shown corresponds to a typical 5% polyacrylamide gel performed for the analysis of 6 potential sites. The positions of proposed cleavage sites are identified at the top of the gel. The negative control performed in the absence of any oligonucleotide is indicated by the letter C.

The initial step in developing a ribozyme capable of specifically cleaving the HCV (+) RNA was the identification of the sites most accessible for targeting. Sites located in single-stranded regions should exhibit a higher ribozyme binding potential than those located in double-stranded regions [33]. In order to address this issue, a bioinformatics approach was initially used to identify the most accessible sites (see Figure 2). Using the RNA folding software RNA Structure 3.7 [34], a series of potential secondary structures that can be formed by the 341-nt RNA sequence were obtained. The twenty most stable structures were then analyzed using the OligoWalk software, which provides, in terms of energy, the degree of accessibility of short sequences along the different structures [34]. A window size of 7 nt, corresponding to the binding domain of the HDV Rz, was used (i.e. the P1 stem). Using a cut-off value of smaller than −12 kcal/mol for the predicted binding constant of the substrate to the ribozyme, this analysis identified 55 potentially accessible sites.

The resulting sites were then further analyzed to see if they fulfilled the criteria essential for efficient HDV Rz cleavage. Specifically, the first nucleotide downstream of the cleavage site (position +1) has to be a guanosine in order to allow formation of a critical GU Wobble bp with the ribozyme (position +1, Figure 1B), and the nucleotide upstream of the cleavage site should not be a guanosine (position −1, Figure 1B). Application of these constraints shortened the list to 15 potential target sites (Figure 2). Next, biochemical assays were performed in order to validate the bioinformatic predictions. Specifcally, RNase H hydrolysis assays were performed in vitro using 7-nt oligonucleotides corresponding to the binding domain of the HDV Rz and a 575-nt HCV-derived RNA species corresponding to the 5′ region of the genome. RNase H specifically cleaves the RNA half of an RNA-DNA duplex. In this analysis, the most accessible sites, that is, those that bind the oligonucleotides, should be cleaved by the RNAse H. Five'-end-labeled HCV-derived transcripts were pre-incubated with the oligonucleotides prior to being incubated with RNase H and analyzed samples. Figure 2 shows an example of autoradiogram performed with a 5% polyacrylamide gel. The time of migration used provides a resolution suitable for the analysis of the sites located between positions 188 to 313. In this example, 5 out of the 6 oligonucleotides generated specific hydrolysis products, although at different levels. It is important to note that because the sites 233 and 311 share the same 7 nt sequence, one oligonucleotide permitted the evaluation at both sites. Efficient hydrolysis was detected at position 311 while only a faith band at position 233. Several electrophoresis with various migration times were performed in order to evaluate the 14 different oligonucleotides. These experiments permitted the validation of 8 potential sites and data was compiled at Table 1. These 8 sites are located throughout the 5′ UTR, albeit with a predominance within the region from 299 to 318 in which four of the eight are located.

**Table 1:** Compilation of the data from the RNase H hydrolysis of HCV (+) RNA.

Cleavage positions	Oligonucleotide sequences	RNase H hydrolysis
15	CGCCCCC	−
110	TGGAGGC	−
117	GGGGTCC	+
176	TCCTGGC	−
184	CCCGGTC	−
188	AGGACCC	−
217	TCCAGGC	−
229	CACGCCC	+
233	GGGGCAC	+
235	CGGGGGC	−
266	GCGACCC	+
299	AGCACCC	+
311	GGGGCAC	+
313	CCGGGGC	+
318	ACCTCCC	+

The site located at position 188 was retained as a control for the subsequent step. The analysis of the 5′ to 3′ distribution of the selected sites revealed a bias that lowered representation in the region spanning positions 1 to 200. Only the site at position 117 was retrieved within this region. In order to obtain more sites in the region spanning positions 1 to 200, 8 other sites were selected. The selection for these new sites was based on the presence of a high level of sequence conservation as well as their potential accessibility according to the reported crystal structure of the HCV IRES [5] (see Table 2). Together, these analyses provided a collection of 17 potential sites for targeting in the HCV (+) strands.

**Table 2:** Compilation of the in vitro cleavage of HCV (+) strand by the SOFA-HDV ribozyme data.

Cleavage positions	Rz P1 sequences	Rz Biosensor sequences	Cleavage %
117	GGGGUCU	UGGCUCUCCCGG	02±1
188	AGGACCU	AUUGAGCGGGUU	02±1
229	CACGCCU	UAGCAGUCUCGC	07±1
233	GGGGCAU	CGGCUAGCAGUC	01±1
266	GCGACCU	GUACCACAAGGC	10±1
299	AGCACCU	CUCCCGGGGCAC	13±3
311	GGGGCAU	GGUCUACGAGAC	07±1
313	CCGGGGU	ACGGUCUACGAG	33±2
318	ACCUCCU	GGUGCACGGUCU	29±1
21	GAGUGUU	GGGAGUGAUCUA	21±1
60	UGAAGAU	GCUAGACGCUUU	39±3
68	UUUCUGU	ACGCCAUGGCUA	35±1
82	CCAUGGU	CGACACUCAUAC	04±1
135	AUGGCUU	CGGUUCCGCAGA	07±1
143	AGACCAU	GUACUCACCGGU	02±1
170	CAAUUCU	AAGAAAGGACCC	01±1
200	UUGAUCU	UCCAGGCAUUGA	04±1

The sequences for each SOFA-HDV Rz corresponding to their P1 and biosensor recognition domains are listed. The upper section includes the SOFA-HDV Rz targetable sites as identified by bioinformatic and biochemical procedures, while the lower section includes those based on the reported secondary and crystal structures.

### Designing of the SOFA-HDV Ribozymes

The various selected target sequences of the HDV ribozymes were all highly conserved among the major HCV genotypes (data not shown), a prerequisite for the development of a viable HDV Rz-based therapeutical approach. Preliminary sequence homology searches between the HDV Rz and a bank of human transcripts revealed that the ribozymes could potentially target several human transcripts (version 35.1 of the human genome). These potential off-target effects resulted from the limited size of the recognition domain present in the original HDV Rz (only 7 nt). It has been estimated that a minimum sequence of 13–14 base pairing nucleotides is required for the specific targeting of an unique RNA species in the human transcriptome [35]. In order to improve the substrate specificity of each ribozyme, a SOFA module was designed for each (see the description of the SOFA in the Introduction section and in reference [29]). In each case the biosensor sequences added are presented in Table 2. The sequence located between the ribozyme and the biosensor binding domains on the HCV target, the so-called spacer domain, was always kept between 4 and 7 nt in length as this length has been shown to be optimal [30], except in the cases SOFA-HDV-Rz170 and -Rz188. The former includes a spacer of 11 nt, while the latter possesses one of 10 nt, and both were required in order to improve the biosensor binding site's conservation level. As a result, the designed SOFA-HDV ribozymes should target only the desired sequence's region, and should therefore be specific enough to target only HCV. Moreover, the addition of the biosensor sequence of the SOFA domain permits the differentiation between the recognition sites of the SOFA-HDV-Rz233 and –Rz311 ribozyme even though they share the same sequence for the P1 region of the ribozyme domain.

### *In Vitro* Cleavage Assay of the Potential SOFA-HDV Ribozymes

**Figure 3:**
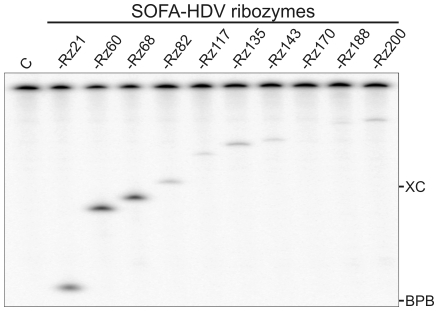
Typical autoradiogram of an 8% polyacrylamide gel performed in order to analyze SOFA-HDV ribozyme cleavage *in vitro*. The experiments were performed using 5′-end-labeled HCV transcripts in the presence of an excess of SOFA-HDV ribozyme. The SOFA-HDV ribozymes are identified at the top of the gel. The negative control performed in the absence of ribozyme is indicated by the letter C. The positions of the xylene cyanol (XC) and bromophenol blue (BPB) marker dyes are indicated.

The next step was the synthesis of a collection of SOFA-HDV ribozymes and the identification of those that demonstrate the highest levels of cleavage activity in vitro. The ability of each SOFA-HDV ribozyme to cleave 5′-radiolabeled 575-nt HCV RNA was tested under single-turnover conditions ([Rz]≫[S]). A typical example is illustrated in Figure 3. The time of migration used for this specific electrophoresis was suitable for the resolution of the sites located in the 5′end of the HCV IRES (positions 1 to 200). In this example, the SOFA-HDV-Rz21, -Rz60 and -Rz68 ribozymes exhibited high levels of cleavage. All others cleaved less efficiently. Five ribozymes exhibited cleavage levels >20%, a level that we considered relatively high for the cleavage of a long and highly structured RNA species. Several electrophoresis using different migration time were performed to monitor the cleavage level at the various positions (raw data not shown) and the data were compiled at Table 2. Overall, 7 out of 17 SOFA-HDV ribozymes exhibited significant cleavage of the substrate (i.e. cleavage percentage >10%; Table 2). Clearly, these results suggest that the time invested in identifying the most accessible sites was productive, as without it this number would certainly have been smaller. The 5 SOFA-HDV ribozymes that exhibited a cleavage level below 2% were discarded at this step. This includes as expected SOFA-HDV-Rz188 that was the negative control. The other 12 SOFA-HDV ribozymes were conserved for the subsequent step.

### Evaluation of the SOFA-HDV Ribozymes Cleaving the HCV (+) Strand In Cellulo

The potential abilites of all SOFA-HDV ribozymes to inhibit an HCV replicon were tested. Prior to this experiment, the ribozymes were cloned adjacent to a tRNA^Val^ in modified pLenti plasmids. The resulting plasmids were used to produce lentivirus which in turn were used to transduce Huh-7 cells (see Materials and Methods). The use of the tRNA^Val^ as both promoter and leader sequence has previously been proven to be successful for both the production and the localization of HDV ribozymes [26]. It permits the production of a large amount of the chimeric RNA by the host RNA pol III. In addition, it is well known that the tRNA motif directs the expressed ribozyme to the cytoplasm, where it hybridizes to the targeted mRNA [36], [37]. The expression of all chimeric tRNA^Val^∶SOFA-HDV ribozymes was confirmed at the time of the replicon inhibition assay (i.e. 48 h post transduction, data not shown). Results from preliminary expression experiments showed that 48 to 72 h post transfection was the optimal window for the expression of the ribozymes. The excess of chimeric tRNA^Val^∶SOFA-HDV ribozymes over targeted HCV strands remained undetermined. However, detection of tRNA^Val^∶SOFA-HDV ribozymes was performed by Northern blot hybridizations and confirmed their relatively large abundance while HCV strand were barely detectable using the same technique (data not shown). These results support the notion that the ribozymes were in excess compared to the HCV substrate, although this does not constitute direct evidence.

**Figure 4:**
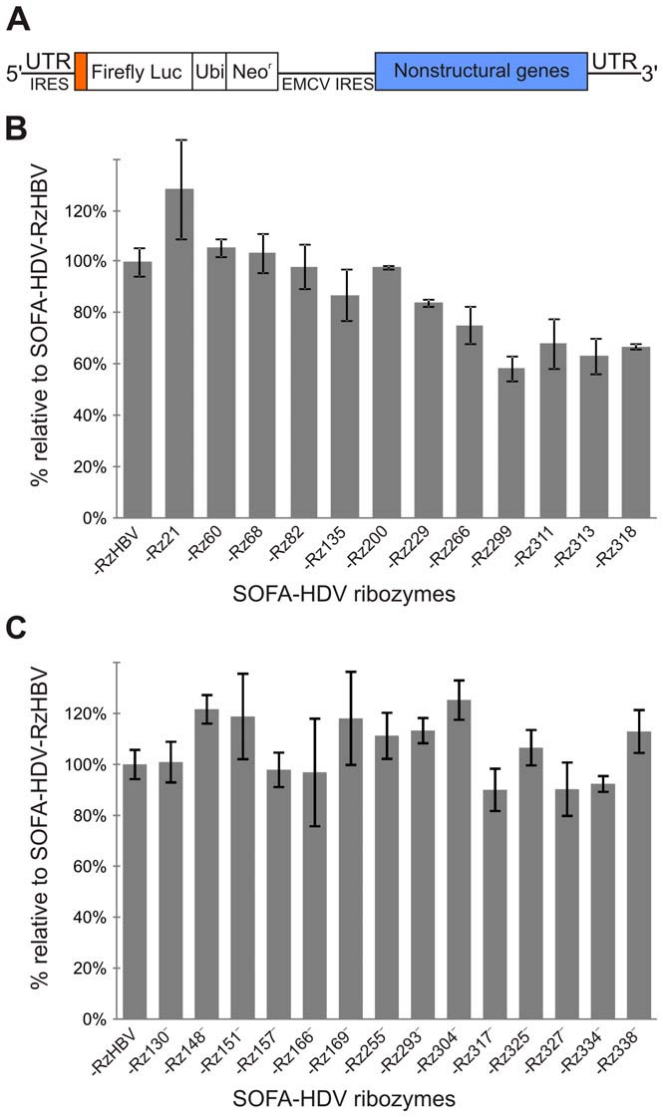
Analysis of the inhibition of the HCV replicon by SOFA-HDV ribozymes. (A) Schematic representation of the HCV replicon used (described previously [38]). (B) and (C). Histograms of the relative luciferase activities detected for the HCV (+) and (−) strands, respectively. Luciferase activity was detected in the presence of all SOFA-HDV ribozymes tested, and was reported relative to that determined for an irrelevant SOFA-HDV ribozyme whose level was arbitrarily set at 100%. The latter SOFA-HDV ribozyme was designed to target the hepatitis B virus and does not possess the sequences required in order to recognize the HCV strands of either the (+) or the (−) polarity. The characterization of this SOFA-HDV ribozyme was previously reported [29].

It is important to note that during an experiment neither G-418 nor antibiotic were used in order to reduce interference with cell transduction as well as to avoid the positive selection of HCV replicon containing cells while measuring the effect of the SOFA-HDV ribozymes. The Huh-7 cells containing the subgenomic biscistronic replicon Luc-ubi-neo-ET, which includes the firefly luciferase ORF that provides a quick enzymatic assay for drug screening, was used (Figure 4A) [38]. Similar replicon systems were used in many other studies in order to screen various nucleic acid-based approaches including siRNA [e.g. 39]–[43]. A colorimetric assay was performed for normalization purposes by considering the total protein concentration. Each SOFA-HDV ribozyme tested was compared to an irrelevant one possessing a recognition sequence that should not be able to interact with either the HCV RNA, nor with any human mRNA (as determined by a homology search). The latter SOFA-HDV ribozyme was originally engineered to target the hepatitis B virus (SOFA-HDV-RzHBV) [29]. Its use permitted determination of the relative percentage of luciferase detected in the presence of each SOFA-HDV ribozyme, as compared to that observed in the presence of the irrelevant one which was arbitrarily set at a luciferase/protein ratio of 100%. At least two or three biological replicates were performed for all ribozymes, and typical results are illustrated in Figure 4B. Simple cell exposition to polybrene, the transduction reagent, or even the expression of the tRNA^Val^ alone, led to a variation of +/−20% (data not shown). Consequently, it was decided that a ribozyme must decrease the luciferase/protein ratio by at least 20% in order for it to be considered a significant inhibition of replication. Using this criteria, 7 SOFA-HDV ribozymes were rejected (i.e. SOFA-HDV-Rz21, -Rz60, -Rz68, -Rz82, -Rz135, -Rz200, and -Rz229). One ribozyme exhibited intermediate inhibition level at 25% (i.e. SOFA-HDV-Rz266). Most importantly, 4 ribozymes exhibited cleavage activities that led to >30% reductions in the luciferase/protein ratio: the SOFA-HDV-Rz311, -Rz313 and -Rz318 reduced the level between 30 and 40%, while SOFA-HDV-Rz299 caused even more significant reduction (i.e. a 42% luciferase/protein).

The experiment was repeated several times, and always produced similar results and experimental variation when the same lentivirus preparation was used, while use of different viral preparations increased the experimental variation (data not shown). The use of different lentivirus concentrations did not result in improved inhibition of the replicon. Similarly, prolonged exposition of the cells to the lentiviruses did not lead to more important reduction of the luciferase activity. In summary, some SOFA-HDV ribozymes exhibited sufficiently high cleavage levels of the IRES that they significantly reduced the luciferase activity; however, the observed level of replicon inhibition was never high enough to warrant further development of the SOFA-HDV ribozyme as an antiviral drug against HCV. In order words, although the new generation of ribozyme, specifically the SOFA-HDV ribozyme, provides a highly specific and improved tool with significant potential for practical application, its potential for controlling HCV replication appears limited.

### Designing SOFA-HDV Ribozymes That Cleave the HCV (−) Strand

One possible explanation for the limited inhibitory effect of the SOFA-HDV ribozyme against HCV (+) strands might be the fact that the replication complex is compartmentalized by a lipid bilayer membrane [32], [44]–[45]. This localization considerably reduces the accessibility of the HCV RNA (+) strand to the SOFA-HDV ribozyme during replication. In fact, the action of the ribozymes may take place mainly, if not exclusively, during the translation of the (+) strand that encodes the polyprotein. If this is indeed the case, it should be almost impossible to target the HCV (−) strand in cellulo because they are non-coding, and, therefore, are not accessible during the translation step. In order to verify this hypothesis, SOFA-HDV ribozymes specifically targeting the 3′-end of the HCV (−) strand that is the counterpart of the 5′ UTR found in the (+) strand were designed and tested for cleavage activity both in vitro and in cellulo.

Instead of applying a complex design strategy, HCV sequence alignment was used to identify both highly conserved sequences in the HCV genome and potential sites for HDV ribozyme cleavage (i.e. those harboring the sequence H_−1_G_+1_; with H being C, U or A). The 3′-end region of the HCV (−) strand was shown to fold into a secondary structure, but not one as stable as the IRES adopted by the HCV (+) polarity strand [46]. Application of this criteria led to the identification of 14 potential sites, all of which were located in the last 341 nt of the HCV (−) strand (see Table 3). The cleavage activities of all of the corresponding SOFA-HDV ribozymes were assayed in vitro using a 575-nt transcript derived from the HCV (−) strand that was the counterpart of the one used for the (+) polarity above. All SOFA-HDV ribozymes exhibited cleavage (>1%, Table 3). Three ribozymes exhibited a limited level of <10%, seven exhibited a significant level of between 10 and 20% and four exhibited a cleavage level over 20%. Specifically, SOFA-HDV-Rz166^−^, -Rz327^−^, -Rz304^−^ and -Rz293^−^ exhibited cleavage activities of 24%, 43%, 70% and 85%, respectively (see Table 3). Observation of such significant levels of cleavage for the HCV (−) strand provides a good indication that it is less stable than its (+) counterpart.

**Table 3:** Compilation of the *in vitro* cleavage of the HCV (−) strand by SOFA-HDV ribozyme data.

Cleavage positions	Rz P1 sequences	Rz Biosensor sequences	Cleavage %
130	CCCUCCU	CAGCCUCCAGG	04±3
148	AGUGGUU	CCUCCCGGGAGA	18±2
151	GGUCUGU	CCCGGGAGAGCC	14±3
157	CGGAACU	AGAGCCAUAGUG	04±3
166	UGAGUAU	GUGGUCUGCGGA	24±5
169	GUACACU	GUCUGCGGAACC	17±1
255	GCUAGCU	GCGUGCCCCCGC	16±7
293	UACUGCU	GCGAAAGGCCUU	85±6
304	AGGGUGU	UUGUGGUACUGC	70±7
317	GUGCCCU	UGAUAGGGUGCU	12±5
325	GGAGGUU	UGCUUGCGAGUG	14±3
327	AGGUCUU	CUUGCGAGUGCC	43±5
334	GUAGACU	GUGCCCCGGGAG	15±5
338	ACCGUGU	CCCGGGAGGUCU	09±3

The sequences for each SOFA-HDV Rz corresponding to their P1 and biosensor recognition domains are listed. Nucleotide positions are numbered from 3′ to 5′ in order to facilitate concordance with the data obtained for the HCV (+) strand.

The cleavage of the HCV (−) strand by all of these SOFA-HDV ribozymes was then accessed using both the replicon and the lentivirus systems as described above. Only 3 SOFA-HDV ribozymes out of the 14 reduced the level of luciferase activity of approximately 10%. Specifically, SOFA-HDV-Rz317^−^, -Rz327^−^ and -Rz334^−^ showed reductions of 10%, 10% and 8%, respectively, all just below the cut-off previously established for considering a ribozyme as being active. In other words, even the ribozymes that possess an outstanding cleavage activity in vitro did not efficiently cleave the HCV (−) in cellulo. Together, these data support the hypothesis that the HCV (−) strand appears to be inaccessible for cleavage by the SOFA-HDV ribozymes.

## Discussion

### Designing SOFA-HDV Ribozymes

We report here the first extensive design of SOFA-HDV ribozymes directed against two distinct RNA targets (considering both the (+) and (−) HCV strands as distinct RNA molecules). Previous studies directed towards the development of a gene-inactivation system based on this nucleic acid approach always included a minimal number of ribozymes (i.e. only one to five ribozymes per target [25], [28], [29]). In the present study, a total of 31 SOFA-HDV ribozymes targeting the HCV strands were designed and their cleavage activities accessed in vitro. Seventeen of these SOFA-HDV ribozymes targeted the HCV (+) strand, while fourteen targeted the (−) counterpart. Although not a very large collection of ribozymes, this number should be sufficient to provide some information on the features that must be considered in the design step. In this report the analysis was limited to the data from in vitro cleavages because inclusion of the in cellulo results implicates too many other important features, including several that remain to be identified.

**Figure 5:**
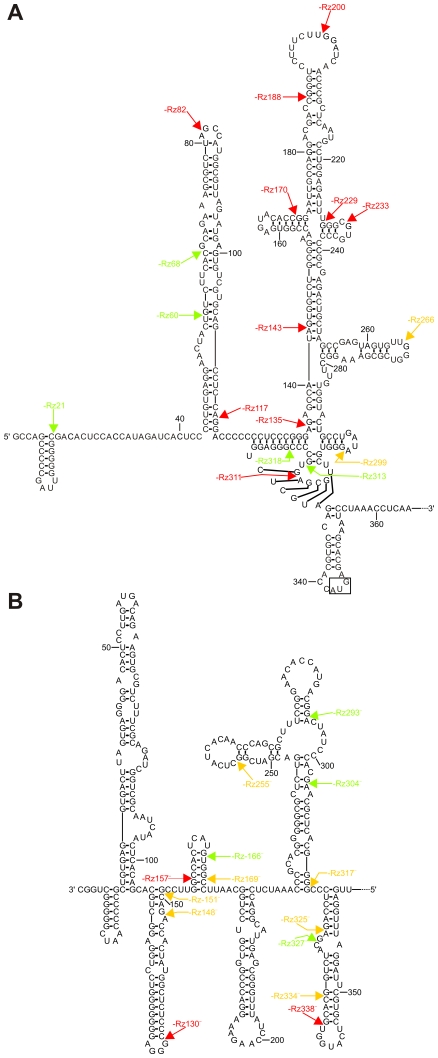
Analysis of the cleavage activity of the SOFA-HDV ribozyme collections. (A) and (B). The cleavage activity levels observed in vitro are illustrated on the proposed secondary structures of both the HCV (+) and (−) strands, respectively. The secondary structures shown are as previously reported [13], [40]. The SOFA-HDV ribozymes exhibiting either no, or relatively low cleavage activities (<10%), are indicated in red. Those exhibiting moderate cleavage activities (between 10% and 20%) are indicated in yellow, while those exhibiting relatively high cleavage activities (>20%) are indicated in green. The arrows indicate the proposed cleavage position of each SOFA-HDV ribozyme.

In order to facilitate the analysis, the cleavage positions of all SOFA-HDV ribozymes tested in vitro are illustrated on the proposed secondary structures of both the (+) and (−) HCV strands (Figure 5). Ribozymes that showed either limited or no cleavage activity are in red, while those exhibiting moderate or relatively high levels of cleavage activity are in yellow and green, respectively.

The structure of a target is obviously an important feature to consider when targeting an RNA molecule in *trans*. The formation of double-stranded RNA within the target region can compete with any ribozyme binding that involves unfavourable intramolecular base pairings. This study presents an elegant example of this situation. In the case of the HCV (+) strand, only 7 out of the 17 SOFA-HDV ribozymes exhibited cleavage activities of greater than 10% (Figure 5A). Six of these ribozymes targeted sites located either side of the highly structured IRES motif. Specifically, 3 SOFA-HDV ribozymes cleaved site before position 68, and 3 after position 299. Conversely, the corresponding region of the HCV (−) strand appears to fold into a less stable secondary structure [46], resulting in 11 out of 14 SOFA-HDV ribozymes that exhibited cleavage activities of over 10% cleaving in this region (Figure 5B). The higher accessibility of the HCV (−) strand, as compared to the (+) strand, also received support from the discovery of two ribozymes that exhibited outstanding cleavage levels when the length of target is taken into consideration (i.e. SOFA-HDV-Rz293^−^ and -Rz304^−^).

The previous elucidation of the secondary structure was of great help in designing the ribozymes directed against both strands. In the case of the HCV (+) strand, the use of bioinformatics coupled to validation by the RNase H hydrolysis was not really more productive, in terms of the number of SOFA-HDV ribozymes that exhibited significant cleavage activity, that was simple analysis of the secondary structure (i.e. 4 and 3 SOFA-HDV ribozymes out of 8 in each case, respectively). However, it is important to remember that the computer-assisted approach coupled to the biochemical validation was performed on the original HDV Rz and only then was the SOFA motif added. It should be noticed that it would be irrelevant to perform RNAse H assays using long oligonucleotides including the complementary sequence for the P1, the spacer and the biosensor sequences. In that case the spacers would also be hybridized favoring significantly the formation of the duplexes, therefore introducing an important bias. Alternatively one might imagine using two distinct oligonuleotides at the same time in the RNase H assay (one for the P1 domain and the other for the biosensor domain). In the later case, the resolution of the electrophoresis would not be sufficient to allow distinction between the binding of each oligonucleotide to the target. However, it has previously been reported that the addition of the SOFA motif reduces the importance of the accessibility hurdle [30]. Most likely the presence of the biosensor domain that forms 11–12 base pairs with the substrate contributes significantly, certainly in terms of binding energy. It is easy to imagine that the subsequent binding of the biosensor and the ribozyme's P1 region to the substrate might occur in a cooperative manner. Finally, we noted that the more accessible the complementary sequence of the biosensor (i.e. the more it is located in a single-stranded region), the more efficient the resulting SOFA-HDV ribozyme tended to be. However, this is not a simple question of adding the base pairs involved in the formation of both the P1 and biosensor domains. For that reason it is complex to consider it during the in silico step of the SOFA-HDV ribozyme' design. There are many factors that influence the binding of a ribozyme to a target that are not trivial to identify. For example there is all the tertiary structure of the ribozyme by itself that will influence the result, which cannot be taken into account and, therefore, may influence the accuracy of the predictions and contributes to some discrepancies.

Observation of the correlation between the sequence composition of the biosensor and the P1 strand of the ribozyme domain, as well as of their binding sequences within the substrate, with the cleavage efficiency prompts several conclusions. With the exception of sequences rich in adenosine and uridine in either of these two domains, these analyses did not reveal any bias towards higher cleavage levels. Moreover, the presence of the biosensor domain seems to reduce the importance of the composition of the P1 domain. Previously reported analyses of HDV ribozyme cleavage using a collection of small substrates revealed that the identity of the base pairs involving the pairing of substrate position +4 with the ribozyme significantly influenced the activity level [47]. For example, the presence of a guanosine residue in the substrate that base paired with a cytosine in the ribozyme resulted in the formation of an unproductive ribozyme-substrate complex. These nucleotides, located in the middle of the P1 stem, have been proposed to be essential for both substrate binding and the subsequent steps in the cleavage pathway [47], [48]. The fact that both the SOFA-HDV-Rz68 targeting the HCV (+) strand, and -Rz169^−^ targeting the HCV (−) strand, each of which possesses a cytosine in this position and exhibits a relatively high level of cleavage activity, suggests that the requirements for the efficient HDV ribozyme targeting of small substrates may differ from those of the SOFA-HDV ribozyme targeting long RNA molecules. Even so, the observed substrate specificity preferences found for positions −1 to −4 adjacent to the cleavage site using a small substrate agree in part with the results observed for the collection of SOFA-HDV ribozymes targeting the HCV strands. Previously, a collection of small substrates possessing both single and multiple mutations in positions −1 to −4 was studied [49]. Some of these substrates were found to be uncleavable, while others demonstrated almost two orders of magnitude of difference, in terms of relative specificity, between the least and the most efficiently cleaved substrates. The nucleotides located at each of these four positions contribute differently to the ability of a substrate to be cleaved [50]. The optimal sequence for positions −4 to −1 was determined to be _−4_YHRH_−1_ (where Y indicates C or U; H indicates A, C or U; and R indicates A or G [49]). Even if this portion of the substrate is not part of the recognition domain (i.e. the base-pairing domain), it has been proposed to play a crucial role as an external determinant of the ability of a substrate to be cleaved in addition to potentially imposing steric hindrances that limit the cleavage activity. Several analyses were performed in order to verify whether or not the cleavage activity of SOFA-HDV ribozymes targeting HCV obeyed the same specificity requirements (raw data not shown). The identities of the bases located in positions −1 and −2 appeared to significantly influence the cleavage level observed. The presence of two consecutive pyrimidines in these positions appears to be detrimental, although to varying degrees. In terms of positions −3 and −4, the nucleotides identities were less important, similar to what is observed for small substrates.

In brief, the cleavage site of a substrate for SOFA-HDV ribozyme should harbor a guanosine in position +1, no guanosine is position −1, should be in agreement with the previously determined preferable nucleotides in positions −1 to −2, and, as much as possible, should be accessible for binding. However, even if in vitro experiments suggest the potential for success, there is no guarantee that this will be the case in vivo. Moreover, there is some, but not a perfect, correlation between the level of cleavage observed in vitro with one detected in vivo. For example, SOFA-HDV-Rz60 exhibited the best cleavage activity of the HCV (+) strand in vitro but it was not the case in cellulo (i.e. 39% in vitro compared to only 5% in cellulo; see Table 2 and Figure 4). Similarly, the best cutter of the HCV (−) strand in vitro was not the one that exhibited the higher level of cleavage in cellulo (i.e. SOFA-HDV-Rz293^−^ exhibited 85% of cleavage in vitro while it was no inhibiting in cellulo). The opposite solution was also observed. For exemple, the SOFA-HDV-Rz299 exhibited only 13% of cleavage in vitro but was the best one in cellulo with a 42% of reduction. There are several parameters in cellulo that may influence significantly the cleavage level and are not occurring in vitro or taken into consideration during the design of the SOFA-HDV ribozyme. Some parameters are related to the target (e.g. the presence of interacting proteins either masking cleavage site or modifying the structure of the substrate) while others are related to the ribozyme by itself (i.e. the turnover and half-life of each ribozymes may differed), to name only these examples. Clearly, this is a complex problem and there is not a simple solution.

### The SOFA-HDV Ribozymes May Be of Limited Interest for HCV Treatment

This report shows that targeting the HCV replicon system using SOFA-HDV ribozymes was not really productive, even when great attention was devoted towards optimizing their design. The in cellulo experiments were repeated several times using different conditions, including the use of various lentivirus preparations, transfection with the ribozyme and use of other HCV IRES-mediated translation systems, to name only a few examples. Even though inhibition of HCV replication was observed, it was only at a relatively limited level. The suitable inhibition level required for further development of a therapeutical approach is at least 80% inhibition. Anything less would likely not lead to viral clearance. Therefore, none of the SOFA-HDV ribozymes examined appeared to be a potential candidate for further development in HCV treatment.

One potential explanation for the limited cleavage activity might be the bicistronic replicon system used to screen the ribozyme in cellulo, although it has been used successfully in many studies [38]–[43]. It was showed that a picornavirus IRES can hand over components of the translation apparatus to another translation start site on the same reporter RNA in *cis*. [51]. By this translation enhancement such an internal picornavirus IRES reduces possible effects of mutations or inhibitors of translation. However, this study did not verify if the reduction of inhibition can be observed when using cleaved mRNA (i.e. resulting from the action of a ribozyme). Therefore, there is no certitude that the camouflage phenomenon may have masked the action of some of the tested SOFA-HDV ribozymes. Moreover, this possibility cannot be evoked in order to explain the low level of cleavage activity observed in cellulo for the collection of SOFA-HDV ribozymes targeting the (−) strand. In that case it has to occur on the opposite strand that is used for the translation.

Another potential explanation might be intrinsic to the SOFA-HDV ribozymes or the fact that the ribozymes were linked to a tRNA for their expression, which may impair their cleavage ability. However, there is increasing evidence that the HCV viral genome replicates in a peculiar membranous structure located around the endoplasmic reticulum [52]–[54]. Electron microscopy observations revealed that cells supporting the replication possess an unusual membranous structure, and that part of the endoplasmic reticulum membrane is notably deformed [44]. This structure has a complicated shape, as well as a botryoidal structure in some cases, and is also seen in liver specimen taken from HCV infected patients. This structure was named a membranous web, and is common to *Flaviviridae*. Both (+) and (−) HCV strands were shown to be synthesized in a membrane-protected complex [44]. Moreover, HCV strands were shown to be resistant to micrococcal nuclease treatment, a treatment that completely hydrolyzed ribosomal RNA [44]. In contrast, HCV strands were fully hydrolyzed by the nuclease treatment only when the experiment was performed in the presence of a detergent which disrupted the membraneous structures. Moreover, it has been shown that the core recruits NS proteins, HCV RNAs and the replication complex to lipid droplet-associated membranes. In fact, it has been shown that lipid droplets play a central role in the production of infectious HCV particles [44], [45]. This situation considerably reduces the accessibility of both the HCV (+) and (−) strands to the SOFA-HDV ribozyme during replication, in agreement with the limited inhibition observed for all of tested SOFA-HDV ribozymes. Furthermore, this fact also explains why other nucleic acid based approaches directed towards controlling the propagation of HCV, including antisense, ribozymes and deoxyribozymes [19]–[22], and for which much effort has been devoted, has yielded no commercial research program to date even though they remains active. It has even been shown that the entire HCV (−) strand was resistant to RNA interference [55]. These studies also support the notion that the cellular localization of the HCV strands significantly limits the possibility of controlling their propagation by targeting the RNA directly, certainly not the (−) strand. In the case of siRNAs designed to target the HCV (+) strands, the best of them achieved ∼60% silencing [11]–[14]. Contrastly, one study did claim viral clearance based on siRNA targeting [15]. One possible explanation for the latest result may be because siRNA take advantage on the cellular protein machinery, which may have for effect to reduce the hurdle of the HCV RNA strand accessibility. Altogether, this collection of data suggests that much more work is required in order to achieve development of a therapeutic approach directed against HCV, including finding strategies that can release the viral strands from their lipid droplets. At least, nucleic acid-based drugs should be tested with an infection system in which the HCV RNA accessibility problem could be overcome. Specifically, replicon containing cells may already possess membranous alterations due to replication complexes. Alternatively, the use of HCV-JFH1 virus cell culture system [41] may provide an open window of time for targeting the HCV within the early stage of replication. When initiating its infection, the viral RNA should be more exposed to cellular translation system in order to produce viral proteins required for RNA replication. At this point, HCV RNA strands should be more available to our targeting component and the resulting inhibition higher.

## Materials and Methods

### SOFA-HDV Ribozymes and DNA Constructs

SOFA-HDV ribozymes were constructed using a PCR-based strategy that included two complementary and overlapping oligonucleotides as described previously [29]. Briefly, two DNA oligonucleotides were used: the universal reverse primer (5′-CCAGCTAGAAAGGGTCCCTTAGCCATCCGCGAACGGATGCCC-3′) and the SOFA-HDV ribozyme sense primer (5′-TAATACGACTCACTATAGGGCCAGCTAGTTT(N)_11–12BS_(N)_4BL_CAGGGTCCACCTCCTCGCGGT(N)_6P1_TGGGCATCCGTTCGCGG-3′), where N represents A, C, G or T and BS, BL and P1 indicate the biosensor, the blocker sequence and the P1 recognition domain, respectively. The SOFA-HDV ribozyme sense primer is specific for each ribozyme and permits the incorporation of the T7 RNA promoter, while the universal antisense primer was used with all ribozymes. The 5′ to 3′ elongation of the DNA sequence that produced a double-stranded DNA template was performed using Pwo DNA polymerase (Roche Diagnostics). The PCR products were ethanol precipitated, dissolved in water and were then used either for in vitro transcription (see below) or in a second PCR reaction (for cloning purposes). The tRNA^Val^∶SOFA-HDV ribozyme chimeric constructs were generated as described previously [26]. Briefly, the first PCR products were used as templates in order to produce a double-stranded DNA sequence containing a KpnI restriction site in 5′. For this second PCR, the universal reverse primer was used in combination with a universal SOFA KpnI forward primer (5′-CGGGGTACCGGGCCAGCTAGTTT-3′) in a filling reaction using Pwo DNA polymerase. This PCR product was ethanol-precipitated prior to digestion with KpnI (New England Biolabs). The digested amplicons were ligated to KpnI/EcoRV co-digested ptRNA^Val^ vector derived from pcDNA3.1 (Invitrogen) as described previously [26]. The resulting constructions produce tRNA^Val^-driven SOFA-HDV ribozymes targeting various regions of either the HCV 5′ UTR or its complementary sequence in the (−) strand. In order to construct lentiviral vectors for each ribozyme, the entire tRNA^Val^∶SOFA-HDV ribozyme was amplified by PCR using purified Pwo DNA polymerase and a tRNA-EcoRI forward primer (5′-TATTGAATTCACCGTTGGTTTCCGTAG-3′) coupled with a SOFA-Rz-XhoI reverse primer (5′-ATAACTCGAGAAAAAAGATCCAGCTAGAAAGGG-3′). EcoRI-XhoI co-digested PCR products and pLentiV5-U6 (Invitrogen) were ligated together, producing a lentiviral vector that expresses the tRNA^Val^∶SOFA-HDV ribozyme (the co-digestion of pLentiV5-U6 removes the U6 promoter). All constructions were verified by DNA sequencing.

Plasmid pHCVA was constructed by cloning the 1348-nt HCV 5′ sequence from pHCV-1b [56] into the HindIII/BamHI sites of pcDNA3 (Invitrogen). Plasmid pHCV(−) was constructed by subcloning the HindIII/KpnI restriction fragment of pHCVA into pcDNA3.1(−) (Invitrogen).

### RNA Synthesis by *In Vitro* Transcription

Both the ribozyme and the HCV-derived transcripts were synthesized by run-off transcription as described previously [29]. Briefly, transcriptions were performed in the presence of purified T7 RNA polymerase (10 µg), RNAGuard (24 U, GE Healthcare), pyrophosphatase (0.01 U, Roche Diagnostics) and either KpnI-linearized pHCVA (5 µg), HindIII-linearized pHCV(−) (5 µg) or PCR product (2 to 5 µM) in a buffer containing 80 mM HEPES-KOH, pH 7.5, 24 mM MgCl_2_, 2 mM spermidine, 40 mM DTT and 5 mM of each NTP in a final volume of 100 µL at 37°C for 2 h. Upon completion, the reaction mixtures were treated with DNase RQ1 (Promega) at 37°C for 20 min, and the RNA then purified by phenol∶chloroform extraction and ethanol precipitation. The resulting pellets were dissolved in equal volumes of ultrapure water and loading buffer (95% formamide, 10 mM EDTA, pH 8.0, 0.025% xylene cyanol and 0.025% bromophenol blue). The samples were then fractionated through either 5% or 8% denaturing polyacrylamide gels (PAGE, 19∶1 ratio of acrylamide to bisacrylamide) in buffer containing 45 mM Tris-borate, pH 7.5, 8 M urea, and 2 mM EDTA. The reaction products were visualized by ultraviolet (UV) shadowing. The bands corresponding to the correct sizes for both the ribozymes and the HCV-derived RNAs were cut out and the transcripts eluted overnight at room temperature in elution buffer (500 mM ammonium acetate, 10 mM EDTA, 0.1% SDS). The transcripts were ethanol precipitated, washed, dried and dissolved in ultrapure water. The RNA was quantified by absorbance at 260 nm.

### RNA Substrate Labeling

In order to generate 5′-end-labeled RNA substrate, 50 pmol of purified transcripts were dephosphorylated by adding 1 U of Antartic phosphatase (New England Biolabs) and incubating for 1 h at 37°C in a final volume of 10 µL containing 50 mM Bis-Propane, pH 6.0, 1 mM MgCl_2_, 0.1 mM ZnCl_2_ and 40 U of RNAGuard. The enzyme was then inactivated by incubating at 65°C for 8 min. The dephosphorylated RNAs (5 pmol) were then 5′-end labeled by incubation for 1 h at 37°C with 3 U of T4 polynucleotide kinase (USB) and 3.2 pmol of [γ-^32P^]ATP (6000 Ci/mmol; New England Nuclear) in the provided reaction buffer. The reactions were stopped by the addition of two volumes of loading buffer. The 5′-end-labeled RNA substrates were fractionated on 5% PAGE gels and the RNAs detected by autoradiography, cut out and eluted as described above.

### Determination of the Most Accessible Sites within the HCV RNA

The selection of potential target sites was performed as previously described [33]. Briefly, the bioinformatic approach includes three steps. First, the RNA Structure 3.7 software was used to both fold the HCV RNA and to predict the most stable secondary structure of the first 341 nucleotides, in terms of energy, of the genotype 1b variant (NCBI database accession number: AJ238799). Secondly, using the OligoWalk software [34], the accessibility for the binding of complementary 7-mer oligonucleotides mimicking the HDV Rz binding domain was assessed in silico on the predicted structures. Thirdly, the accessibility of the resulting sites in vitro was verified by ribonuclease H probing. The reactions were performed using 5′-end-labeled HCV-derived 575-nt transcripts (∼0.1 µM, ∼50,000 CPM) in combination with 7-mer oligonucleotides (5 µM) complementary to a specific sequence on the substrate. Each oligonucleotide was used in the presence of the substrate, and was pre-incubated for 10 min at 25°C in a final volume of 8 µL containing 20 mM Tris-HCL, pH 7.5, 20 mM KCl, 10 mM MgCl_2_, 0.1 mM EDTA and 0.1 mM DTT. RNase H (0.5 units, Ambion) was then added (2 µL) and the samples were incubated at 37°C for 30 min. The reactions were quenched by the addition of one volume of loading buffer, loaded on denaturing 5–10% PAGE gels and, after electrophoresis exposed to PhosphorImager screens (Storm apparatus, Molecular Dynamics).

### *In Vitro* Ribozyme Cleavage Assays

The conditions for SOFA–HDV ribozyme cleavage assays have been described previously [29]. Briefly, 5′-end-labeled 575-nt HCV-derived transcripts of 575 nt (∼50,000 CPM) were mixed with SOFA-HDV Rz (10 pmol) in a 20 µL reaction containing 50 mM Tris-HCl, pH 7.5 and 10 mM MgCl_2_, and were then incubated at 37°C for 3 h. The reactions were stopped by the addition of one volume of loading buffer, fractionated on denaturing 5–8% PAGE gels which were then exposed to PhosphorImager screens.

### Lentivirus Production, Cell Culture and In Cellulo Assays

Lentivirus were produced as described previously [57]. Briefly, 5×10^6^ HEK 293-FT cells were seeded in 100 mm tissue culture dishes. The next day, the media was replaced by Opti-MEM (Gibco-BRL) media prior to transfection of 6 µg each of pLenti tRNA^Val^∶SOFA Rz, pLP1, pLP2 and pLP/VSVG (Invitrogen) with Lipofectamine 2000 (Invitrogen). After a 4 h incubation, the Opti-MEM was changed for DMEM containing 10% FBS. At 72 h post-transfection, the viral supernatant was filtered through a 0.45 µm Millipore filter. Each viral stock was titrated by counting the number of CFU produced by transduction of HT1080 cells with different dilutions of the viral stock.

Huh-7 cells containing the subgenomic biscistronic replicon Luc-ubi-neo-ET (kindly provided by R. Bartenschlager [37]) were cultured in 100 mm cell culture dishes in Dulbecco's Modified Eagle Medium (DMEM, Wisent) supplemented with 10% FBS (Wisent), 1 mM sodium pyruvate, 2 mM L-glutamine, 250 µg/mL G-418 (Wisent). The cells were passaged twice weekly at a dilution of 1∶4-1∶8 depending on the rate of cell growth. Prior to transduction, Huh-7 cells containing the bicistronic replicon were seeded in 24 well plates at a confluence of 5×10^4^ cells per well. From that point and until the end of the experiment, Huh-7 cells containing the replicon were never in contact with media containing G-418 or antibiotic. The next day, the media was replaced by 250 µL of filtered lentivirus stock (MOI≫1) containing 6 µg/mL hexadimethrine bromide (polybrene, Sigma Aldrich) and the plates then incubated at 37°C for 48 h.

The dual-luciferase reporter assay system (Promega) was used in order to monitor the firefly luciferase as reported elsewhere [58]. Briefly, the cells were washed with 1X PBS and then were lysed in 150 µL of Passive Lysis Buffer for 20 min at room temperature. The luciferase activity was measured by the addition of 20 µL of lysate to 100 µL of luciferase assay reagent II (Promega) in a 5 mL test tube followed by reading on a Berthold Lumat LB9501 luminometer (Berthold Technologies). For each lysate, the total protein concentration was determined using the Bradford assay (Bio-Rad). Data was expressed as a ratio of RLU per µg of total protein, and was then normalized for each ribozyme by dividing by the result obtained with a control ribozyme (i.e. SOFA-HDV-RzHBV). Both the mean value and the standard deviation were calculated for each ribozyme.
